# Comparison of attitudes toward disability and people with disability among caregivers, the public, and people with disability: findings from a cross-sectional survey

**DOI:** 10.1186/s12889-016-3670-0

**Published:** 2016-09-29

**Authors:** Qiaolan Zheng, Qi Tian, Chun Hao, Jing Gu, Jianting Tao, Zuoyi Liang, Xinlin Chen, Jiqian Fang, Jianhua Ruan, Qiuxiang Ai, Yuantao Hao

**Affiliations:** 1 Department of Medical Statistics and Epidemiology, School of Public Health, Sun Yat-sen University, Guangzhou, Guangdong People’s Republic of China; 2 Guangzhou Service Center of Assistive Devices, Guangzhou, Guangdong People’s Republic of China; 3 Guangzhou Disabled Person’s Federation, Guangzhou, Guangdong People’s Republic of China; 4 Department of Preventive Medicine and Health Statistics, College of Fundamental Medical Science, Guangzhou University of Chinese Medicine, Guangzhou, Guangdong People’s Republic of China

**Keywords:** Attitude(s) toward disability, People with disability, Caregivers, Public

## Abstract

**Background:**

A negative attitude toward disability is one of the potential barriers for people with disability (PWD) to achieve social equality. Although numerous studies have investigated attitudes toward disability, few have evaluated personal attitudes toward disability among PWD, and made comparisons with attitudes of healthy respondents. This study was to investigate and compare the attitudes of PWD, caregivers, and the public toward disability and PWD in China, to identify discrepancies in attitude among the three groupsand to examine potential influencing factors of attitude within each group.

**Methods:**

A cross-sectional study was conducted among 2912 PWD, 507 caregivers, and 354 members of the public in Guangzhou, China. Data were collected on participants’ socio-demographic information and personal attitudes toward disability using the *Attitude to Disability Scale* (ADS). ANOVA and ANCOVA were applied to compare the level of attitude among the three groups. Simple and multiple linear regression analyses were used to investigate the relationship between each background factor and attitude within each group.

**Results:**

Over 90 % of caregivers were PWD’s family members. After controlling the socio-demographic characteristics, caregivers had the lowest total scores of ADS (caregivers: 47.7; PWD: 52.3; the public: 50.5). Caregivers who had taken care of PWD for longer durations of time had a more negative attitude toward disability. In contrast, PWD who had been disabled for longer times had a more positive attitude toward disability.

**Conclusions:**

The current national social security system of China does not adequately support PWD’s family-member caregivers who may need assistance coping with their life with PWDs. More research is needed, and the development of a new health-care model for PWD is warranted.

## Background

Disability has become a natural part of the human condition due to population ageing, the increase of chronic diseases, and medical advances that preserve and prolong life. Globally, adding life to years has become as important as adding years to life and is now on the agenda of the United Nations’ Millennium Development Goals (MDG) [1]. According to the report from the World Health Organization (WHO), there are over one billion people estimated to be living with disability in the world [2]. Social inclusion and community participation of people with a disability (PWD) are a central concept guiding current policies for disabled persons around the world [2]. As nations are realizing, negative attitudes toward disability affect the integration of disabled persons into the community [3, 4], and thus may incur the loss of a potential resource. Negative perceptions can lead to lack of opportunities and work, low self-esteem, and isolation, and consequently to stigmatization, marginalization, and recurring negative health outcomes that prolong the discomfort of PWDs and also create a substantial social burden [5–7]. Identifying and understanding negative attitudes toward disability can helpfurther define the factors that hinder or foster PWD’s health and social integration, as well as the development and effectiveness of necessary corollary services. While much research on attitudes toward disability has been conducted in developed countries [8], scant research has been undertaken in developing countries. By the end of 2010, the number of PWD in China amounted to more than 85 million [9], yet little is known about attitudes toward disability even though a substantial national burden of disability exists.

In health and medicine, attitudes toward disability are defined as the cognitive and behavioral processes that involve judgment and favorable/unfavorable reactions to aspects of disability [10]. One recurrent focus of research on attitudes toward disability has been the attitude of the general public [11, 12], and as a result it is well documented that negative public attitudes foster low expectations, discriminatory behaviors, and marginalization of PWD, whereas positive attitudes lead to acceptance of PWD and promote integration into society [2, 13]. In traditional Chinese culture “disability is viewed as a punishment for the disabled person’s sins in a past life or the sins of the person’s parents” [14, 15]. With more urban and younger populations, attitudes may be changing but because reliable data are unavailable, the extent and influence of this perception is not well established. Thus, this study investigated public attitude to detect and measure perceptions and influences extant in the general population that are hindering and fostering PWD’s health and social integration in the country.

Caregivers are a second focus of most research on attitudes to disability. Due to China’s collectivist culture, family members play a significant role in providing care and support for PWD at the family level [14]. In prevailing national conditions with a substantial lack of support in the community, PWDs have an increased dependence on their caregivers. Although caregivers do not experience disability themselves, they have to cope with multiple and conflicting related responsibilities: with the disability-related physical and emotional problems and practical medical care of PWD, with their own individual problems, and with family roles and relations. With a dual role as caregiver and close associate/relative to a person with a disability and his/her family, the attitude of caregivers to disability is influenced by the disability as well. A number of studies have reported the courtesy stigma exhibited by caregivers of PWD [16–18]; however, because this stigma is only one component of a complex of attitudes, few studies reported PWD family-member caregivers’ attitudes in Chinese society. Generally, a negative attitude can undermine the quality of assistance and support, thus decreasing PWD’s quality of life [19] and inhibiting PWD integration into the community. Lower prospects for the disabled may lead to maladaptive coping and inconsistent rehabilitation or treatment [20] and greater strain on both PWD and caregivers. Disability-related stigma or discrimination may interfere with a caregiver’s seeking help [17, 18, 21] to cope with increasing stress. But little has been established about whether or how a courtesy stigma may be bearing influence upon this group, and reliable data on caregiver attitudes toward disability and PWD in China were not available.

Consequently, the gap in research devoted to understanding the attitude to disability and PWD particularly among family-member caregivers in China helped initiate this investigation.

A third group focus consists of people with disabilities themselves. PWD’s attitude toward their own disability is significantly shaped by the experience of social interaction [22]. According to our previous study [19], PWD’s positive attitudes toward disability were highly associated with improved quality of life. Understanding PWD’s attitude toward their disability is a first step in the development of effective behavioral intervention as positive attitudes may result in corresponding positive behaviors [23]. Therefore, knowledge about PWD’s attitude could help improve relevant health services and facilitate PWD’s self-acceptance, fostering integration into society [13]. Most existing research assessing attitudes toward disability has targeted the public, caregivers, or health professionals separately [8], and consequently this lack of data on PWD’s attitude toward their disability also helped initiate this study.

As stated, a purpose of this investigation was to identify any existing discrepancies in attitudes toward disability among the public, PWD, and caregivers. It is well known that attitudes can be formed from a person’s past and present experiences [24]. The general public’s experiences related to disability may result from television, social media, or temporary contact with PWD, whereas PWD’s and caregivers’ experiences originate in their personal daily lives coping with disability. While a large share of research has assessed the attitude of the general public to reflect the level of social inclusion, it is to be noted that the degree of inclusion of or discrimination against PWD perceived by the general public and the caregiver/PWD can vary greatly. For example, the general public may perceive more inclusion and less discrimination against PWD, but PWD/caregivers with immediate experience of disability may perceive the opposite to be true. The difference of attitudes has not been sufficiently examined, and as a result one goal of our study was to identify discrepancies which could suggest the gaps between attitudes perceived by the public and PWD/caregivers who are living in the same society and hint at whether community participation-related interventions would be enhanced among caregivers/PWD.

Differences in the type of disability-related activities PWD and caregivers perform, the perceived difficulty of caregiving for daily living tasks, emotional and nonmedical needs, and so forth, suggest that attitudes toward disability and PWD also vary among PWD and caregivers [25, 26]. The important disparities in the attitudes of these two groups must be addressed to develop tailored educational interventions for PWD and caregivers that foster hope, positive attitudes, and healthy individual and cooperative behaviors for coping with disability and contributing toward improved PWD social inclusion and better quality of life for PWD and their caregivers.

The primary purpose of this study was to investigate the attitudes of all three groups— PWD, caregivers, and the public— toward disability in China and to compare these attitudes among the three groups. To accomplish this, we used the global assessment *Attitudes to Disability Scale* [27] developed by the World Health Organization Quality of Life-Disability Group (WHOQOL-DIS) in order to identify discrepancies in attitudes. The potential influencing factors of the attitudes about disability were also examined within each group. The hypothesis of this study is that the attitudes toward disability and PWD differ among the three groups, and that among them PWD may not hold the most negative attitude toward disability.

## Methods

### Recruitment and participants

Some contents of our study methods have been previously described [19]. Briefly, from March to August 2008, a cross-sectional survey was conducted in Guangzhou, the capital of Guangdong Province in southern China. Guangzhou has 8 million permanent residents, 60 % of whom reside in urban areas and 40 % in suburban areas [28]. Of Guangzhou’s permanent residents, 5.86 % live with some form of disability [29]. The sampling frame of PWD in this study was restricted to all PWDs who held the Disabled Person Card (DPC) in Guangzhou. The DPC, which is issued and managed by the Disabled Persons’ Federation (DPF), is PWD’s permit to access disability benefits and allowances [30]. PWD in this survey were recruited with a three-stage sampling. According to the ratio of urban and suburban populations in Guangzhou, the first stage involved a random selection of three urban districts and two suburban districts from the total of the 12 districts of Guangzhou. The second stage involved a random selection of three sub-districts from each district, generating 15 sub-districts. At the final stage, four communities per sub-district were randomly selected; 60 communities were then finally chosen. All PWD residing in the selected communities were invited to participate in this study. Eligibility criteria of PWD included being a Guangzhou permanent resident, aged 18 or above, and being legally certified as disabled which is operationalized as those PWD with the DPC. Furthermore, eight caregivers of the abovementioned PWD were randomly selected and recruited from each community. For the general public, six general residents were recruited from each selected community as well. Eligibility criteria for each group were as follows. For caregivers, each participant had to be aged 18 or above, have more than 1 year of experience in taking care of a person with a disability, and have no form of disability him- or herself. Participants chosen from the general public had to be aged 18 or above and neither PWD nor caregivers. The response rates were 99.2, 99.8, and 88.5 % among PWD, caregivers, and the public, respectively. Overall, 2912 PWD, 507 caregivers, and 354 members of the public were recruited in the survey.

A DPF staff member and a doctor confirmed eligibility and obtained informed consent from participants. The questionnaire was administered in person by an experienced research interviewer in a private room in each community. The interview was conducted in Chinese and took around 10 min. All the participants were given a gift for their participation. The study protocol was approved by the Institutional Review Boards of Sun Yat-sen University and Guangzhou DPF.

### Measures

Three questionnaires were prepared for the PWD, caregivers, and the public, respectively. Each questionnaire had two parts:

- *Part I* recorded personal information: gender, age, education, and marital status for every respondent; occupations, income levels, types of disability, disability duration, disability visibility, and comorbidity (comorbid with other health problems, including musculoskeletal problems, cardiovascular diseases, respiratory problems, neuropsychological problems, digestive problems, diabetes, sensory organ damage, cancer, and others) among PWD respondents; duration of care-giving and relationship to the PWD among caregivers respondents.
- *Part II* was the ADS. This scale has been verified as accurate in the global assessment of attitudes toward disability and people with disability in both disabled people and in healthy respondents [27]. It is also valid for versions in different languages, including Chinese [31].

The ADS [31] assessed personal attitudes toward disability and people with disability. The 16-item measure was developed by the WHOQOL-DIS [27]. The scale includes the “personal” set of questions for the PWD and “general” set of questions for those without disability. The two sets have the same questions and scoring method but different forms of personal pronouns. PWD’s questions used the first person (*I*) while the caregivers and the public used the third person (*he/she/they*). The measure was scored on a five-point Likert scale, and the total score (range: 16–80) was based on a summation of all 16 items. A higher total score indicates more positive attitudes. Attitude toward disability was explained in four domains: *Inclusion* (relationships, inclusion, burden to society, burden to family), *Discrimination* (ridicule, exploitation, irritation, ignorance), *Gains* (emotional strength, maturity, achievement, determination), and *Prospects* (sexuality, underestimation, optimism, future prospects) (Table 2). Higher mean scores for each domain were indicative of better inclusion, less discrimination, more gains, and better prospects. The total Cronbach’s alphas in the current study were 0.77; the Cronbach’s alphas of the domain were 0.76, 0.74, 0.75, and 0.72, respectively.

### Statistics analysis

Each domain and the total score of the ADS among the three groups was calculated. One-way ANOVA was applied to compare the domains and total scores of the three groups (the variances were equal among the three groups), and ANCOVA was used to compare the domains and total scores of the three groups after controlling for gender, age, education, and marital status [32]. Bonferroni correction was applied for multiple-group comparisons. Within each group, simple linear regression analysis was used to investigate the relationship between the ADS and each background factor (gender, age, education, and marital status), which was intended to present the difference in the ADS among participants with different background factors. Other than background factors, simple and multiple linear regression analyses were also used to investigate the relationship between the ADS and occupations, income levels, types of disability, disability duration, disability visibility, and comorbidity among PWD, as well as ADS and duration of care-giving and relationship to the PWD among caregivers. Multiple linear regression analysis here was applied to investigate the effects of other factors (employment, comorbidity, types of disability, etc.) on ADS after adjusting the effects of background factors (gender, education, and marital status). The exclusion of age in the multiple linear regression analysis was due to collinearity occurring when age was in the model.

Data were double-entered and cross-checked using the EpiData software (EpiData 3.1 for Windows, The EpiData Association Odense, Denmark). Statistical Product and Service Solutions was used for data analysis (SPSS 20.0 for Windows, IBM Corp, USA). Statistical significance was defined by *p* value <0.05.

## Results

### Social-demographic characteristics of the participants

Sociodemographic characteristics (age, gender, education, and marital status) broken down by the three groups are presented in Table 1. All group differences were statistically significant (*p* < 0.001).

**Table 1:** Social-demographic characteristicof the participants

Groups	People with disability *n*_1_ = 2912	Caregivers *n*_2_ = 507	Public *n*_3_ = 354	*χ* ^2^ */H*	*p*
Gender, *n* (%)					
Female	1167 (40.1)	279 (55.0)	179 (50.6)	53.0	<0.001
Male	1745 (59.9)	228 (45.0)	175 (49.4)		
Age, *n* (%)					
18~	164 (5.6)	22 (4.3)	112 (31.6)	55.5	<0.001
30~	410 (14.1)	55 (9.9)	43 (12.1)		
40~	903 (31.0)	136 (26.8)	52 (14.7)		
50~	859 (29.5)	135 (26.6)	40 (11.3)		
60~	568 (19.5)	162 (32.0)	105 (29.7)		
Missing	8 (0.3)	2 (0.4)	2 (0.6)		
Education, *n* (%)				205.2	<0.001
Illiterate	366 (12.6)	43 (8.5)	13 (3.7)		
Primary school	965 (33.1)	169 (33.3)	65 (18.4)		
Secondary school	1520 (52.2)	238 (46.9)	144 (40.7)		
College and above	55 (1.9)	45 (8.9)	126 (35.6)		
Missing	6 (0.2)	12 (2.4)	6 (1.7)		
Marital status, *n* (%)				105.4	<0.001
Single/Widowed	976 (33.5)	55 (10.8)	112 (31.6)		
Married/cohabiting	1932 (66.3)	451 (89.0)	241 (68.1)		
Missing	4 (0.1)	1 (0.2)	1 (0.3)		

### Comparison of attitudes toward disability among PWD, caregivers, and the public

**Fig. 1:**
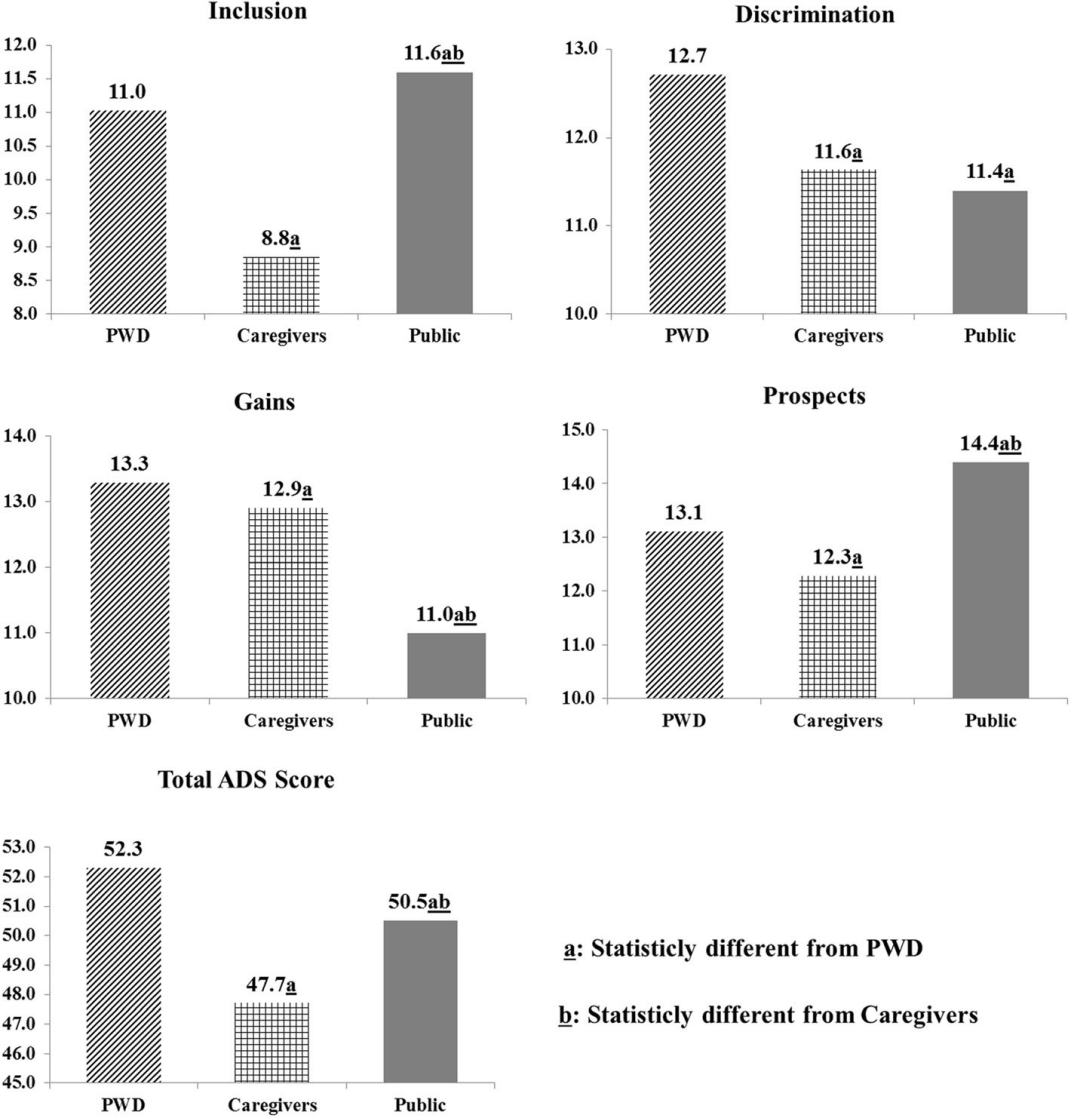
Comparison of attitude to disability after controlling for socio-demographic characteristics (gender, age, education, and marital status) among PWD, caregivers, and the public

As described in Fig. 1, after controlling the socio-demographic characteristics (age, gender, education, and marital status), PWD had the highest total scores of ADS (52.3), significantly higher than caregivers (47.7) and the public (50.5). Caregivers’ total score of ADS was the lowest among the three groups.

For specific domains, caregivers again showed the lowest scores in the domains of *Inclusion* (8.8) and *Prospects* (12.3). Furthermore, PWD showed the highest scores in the domains of *Discrimination* (12.7) and *Gains* (13.3). The public was the group with the lowest scores in *Gains* (11.0), and caregivers were in the middle (12.9) (Fig. 1). The comparisons of each item across the three groups are tabulated in Table 2.

**Table 2:** Comparison of attitudes to disability among PWD, caregivers, and the public

Domain/Item	PWD	Caregivers	Public	*F*	*p*
Inclusion^abc^	11.0 ± 3.0	8.8 ± 2.9	11.8 ± 3.2	134.6	<0.001
Relationships^ac^: PWD find it harder than others to make new friends	2.9 ± 1.0	2.2 ± 1.0	2.9 ± 1.2	87.6	<0.001
Inclusion^abc^: PWD have problems getting involved in society	2.8 ± 1.0	2.0 ± 0.9	2.5 ± 1.0	119.6	<0.001
Burden society^abc^: PWD are a burden on society	2.9 ± 1.0	2.5 ± 1.1	3.4 ± 0.9	84.3	<0.001
Burden family^abc^: PWD are a burden on their family	2.5 ± 1.0	2.1 ± 0.9	3.0 ± 1.1	87.5	<0.001
Discrimination^ab^	12.7 ± 3.0	11.6 ± 3.1	11.6 ± 2.3	45.7	<0.001
Ridicule^abc^: people often make fun of disabilities	3.0 ± 1.0	2.7 ± 1.1	3.5 ± 1.1	64.2	<0.001
Exploitation^ac^: PWD are easier to take advantage of (exploit or treat badly) compared with other people	3.1 ± 1.0	2.8 ± 1.1	3.1 ± 1.1	14.1	<0.001
Irritation^abc^: people tend to become impatient with those with a disability	3.2 ± 0.9	2.9 ± 1.1	2.4 ± 0.9	121.7	<0.001
Ignorance^abc^: people tend to treat those with disability as if they have no feelings	3.4 ± 0.9	3.2 ± 1.0	2.5 ± 0.9	170.3	<0.001
Gains^bc^	13.3 ± 2.5	13.0 ± 2.8	11.2 ± 2.1	106.1	<0.001
Emotional strength^abc^: having a disability can make someone a stronger person	3.6 ± 0.8	3.5 ± 0.9	2.7 ± 1.0	169.0	<0.001
Maturity^bc^: having a disability can make someone a wiser person	3.1 ± 0.9	3.0 ± 0.9	2.2 ± 0.8	205.8	<0.001
Achievement^bc^: some people achieve more because of their disability	3.3 ± 0.8	3.2 ± 0.8	3.4 ± 0.9	4.2	0.015
Determination^bc^: PWD are more determined than others to reach their goals	3.3 ± 0.8	3.2 ± 0.8	3.0 ± 0.8	26.7	<0.001
Prospects^abc^	13.1 ± 2.5	12.3 ± 2.7	14.6 ± 2.5	87.0	<0.001
Sexuality^abc^: sex should not be discussed with PWD	3.5 ± 0.7	3.3 ± 0.7	3.6 ± 0.9	17.3	<0.001
Underestimation^abc^: people should not expect too much from PWD	3.3 ± 0.9	3.1 ± 1.0	3.7 ± 0.9	41.6	<0.001
Optimism^abc^: PWD should not be optimistic (hopeful) about their future	3.3 ± 0.9	3.1 ± 1.0	4.0 ± 0.9	113.0	<0.001
Future prospects^abc^: PWD have less to look forward to than others	3.1 ± 0.9	2.7 ± 0.9	3.3 ± 0.9	50.5	<0.001
Total^ac^	50.1 ± 7.1	45.8 ± 7.5	49.2 ± 5.4	83.2	<0.001

Bonferroni correction was applied for multiple comparison, ^a^significant difference between PWD and caregiers; ^b^significant difference between PWD and public; ^c^significant difference between caregivers and the public

### Sub-group analysis: total ADS scores on characteristics

**Fig. 2:**
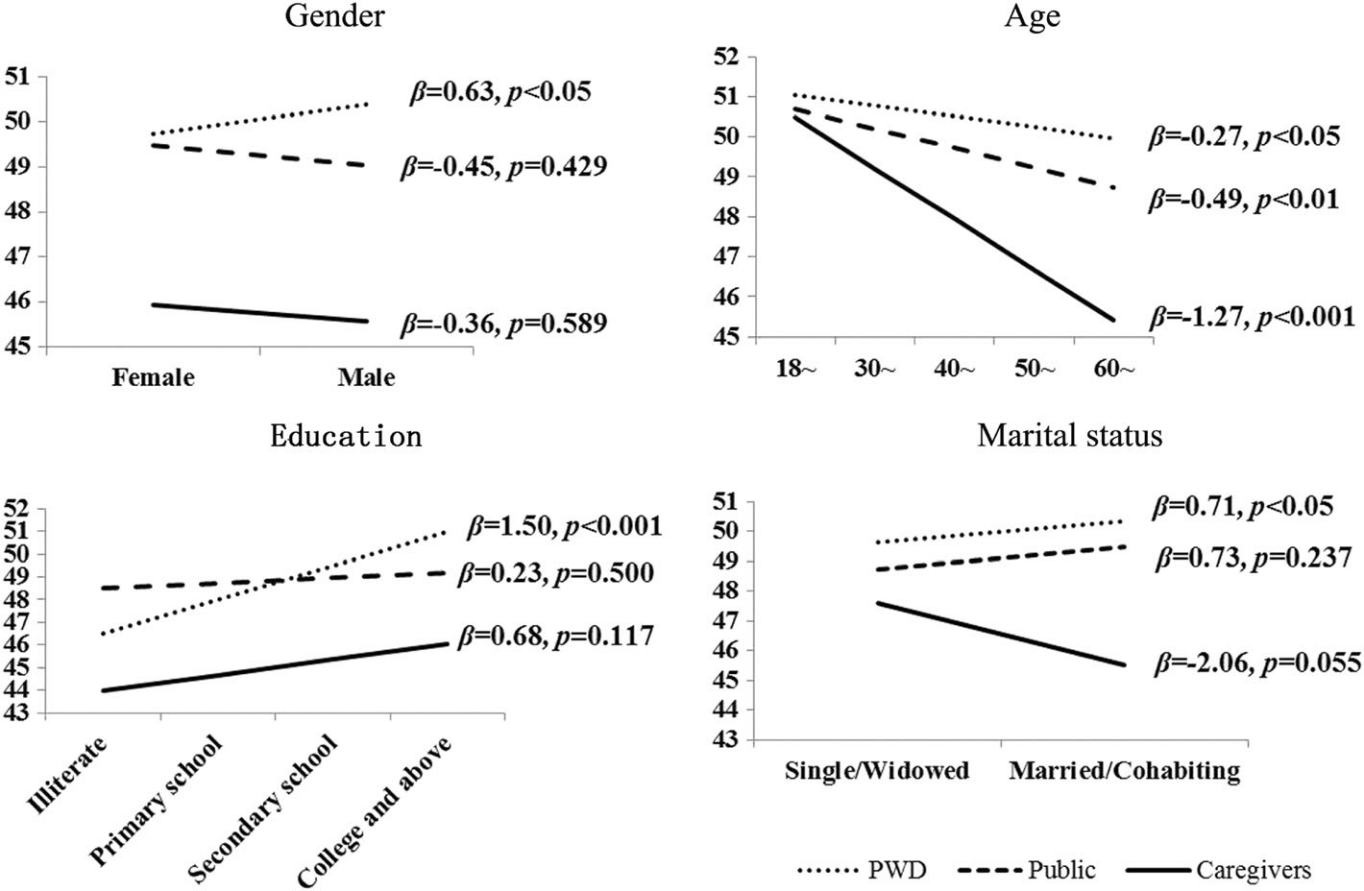
Simplelinear regression analysis to investigate the difference of attitudes to disability depending on socio-demographic characteristics within each group of participants

The results are shown in Fig. 2 and Table 3. The effects of age on ADS were significant and the coefficients were negative within all three groups, indicating that no matter whether the group was PWD (beta = −0.27, *p* = 0.025), caregivers (beta = −1.27, *p* < 0.001), or the public (beta = −0.49, *p* = 0.005), those who were older held a more negative attitude toward disability (Fig. 2). For PWD, those who were male (beta = 0.63, *p* = 0.021) with a higher educational level (beta = 1.50, *p* < 0.001) or currently married (beta = 0.71, *p* = 0.011) had a more positive attitude toward disability (Fig. 2). No significant association was found between gender, educational level, marital status, and ADS within either the groups of caregivers or the public (Fig. 2).

**Table 3:** The difference of ADS depending on characteristics of PWD and caregivers

Groups	n (%)	Attitude (M ± SD)^a^	*B*	*p*	*B*_*a*_	*p*_*a*_
Among PWD (*n* = 2912)						
*Income level* ^b^						
Below average	2329 (80.0)	49.9 ± 6.9	Reference			
No less than average	577 (19.8)	51.2 ± 7.8	1.3	<0.001	1.2	<0.001
*Employment*						
Unemployed	2084 (71.6)	49.6 ± 6.9	Reference			
Retired	333 (11.4)	51.6 ± 7.3	2.0	<0.001	1.5	<0.001
Employed	462 (15.9)	51.2 ± 7.8	1.5	<0.001	1.4	<0.001
Others	30 (1.0)	49.1 ± 9.3	−0.6	0.674	0.6	0.636
*Comorbidity*						
No	2229 (76.5)	50.1 ± 7.3	Reference			
Yes	682 (23.4)	50.3 ± 6.6	−0.2	0.358	−0.1	0.701
*Type of disability*						
Multiple disability	31 (1.1)	46.1 ± 10.4	Reference			
Mental disability	513 (17.6)	49.7 ± 6.4	3.5	0.007	3.0	0.022
Physical disability	1853 (63.6)	50.2 ± 7.4	4.1	0.002	3.7	0.004
Hearing/speech disability	201 (6.9)	50.3 ± 6.6	4.2	0.002	4.3	0.002
Visual disability	314 (10.8)	50.7 ± 6.6	4.5	0.001	4.3	0.001
*Disability duration*						
0~	500 (17.2)	49.1 ± 7.2	Reference			
10~	542 (18.6)	50.1 ± 7.1	1.0	0.018	0.9	0.048
20~	399 (13.7)	50.0 ± 6.9	0.9	0.048	0.9	0.057
30~	371 (12.7)	50.3 ± 7.3	1.2	0.014	1.2	0.011
40~	582 (20.0)	50.5 ± 6.9	1.4	0.001	1.1	0.009
50~	461 (15.8)	50.5 ± 7.0	1.4	0.003	1.4	0.002
*Disability visibility*						
Totally	719 (24.7)	48.2 ± 7.9	Reference			
Mostly	520 (17.9)	49.7 ± 7.1	1.5	<0 .001	1.4	0.001
Moderately	1021 (35.1)	50.5 ± 5.9	2.3	<0.001	2.1	<0.001
A little	349 (12.0)	51.4 ± 7.9	3.2	<0.001	3.0	<0 .001
Not at all	259 (8.9)	52.7 ± 6.6	4.5	<0.001	4.3	<0 .001
Among caregivers (*n* = 507)						
*Relationship with the PWD*						
Parents/Children	278 (54.8)	45.1 ± 7.1	Reference			
Spouse	132 (26.0)	46.8 ± 7.6	1.6	0.039	1.9	0.018
Sibling	46 (9.1)	46.9 ± 7.6	1.8	0.130	1.7	0.144
Others	46 (9.1)	46.4 ± 7.6	1.2	0.285	0.9	0.452
*Caring duration*						
40~	25 (6.9)	43.1 ± 7.0	Reference			
30~	57 (11.2)	44.2 ± 8.4	1.1	0.499	0.4	0.801
20~	115 (22.5)	45.7 ± 6.8	2.6	0.070	2.2	0.132
10~	160 (31.6)	46.6 ± 7.2	3.5	0.011	3.3	0.022
0~	138 (27.2)	46.2 ± 7.7	3.1	0.026	2.8	0.054

*M, SD* mean and standard deviation, *B* unstandardized coefficient in simple linear regression analysis, *B*_*a*_, *p*_*a*_ unstandardized coefficient and *p* value after controlling for socio-demographic characteristics (gender, education, and marital status). ^a^Score range 16–80; ^b^the per capita income of urban and suburban residents in Guangzhou were $4300 and$1700, respectively

With regard to disability duration, compared with PWD who had been disabled less than 10 years (ADS = 49.1), those who had been disabled for longer periods had a more positive attitude toward disability (50.0 - 50.5); whereas caregivers who had taken care of PWD for longer durations of time had a more negative attitude toward disability (Table 3). PWD who were employed (51.2 *vs*. 49.6) and with higher income levels (51.2 *vs*. 49.9) also had a more positive attitude toward disability. In addition, PWD’s ADS scores decreased with their levels of disability visibility (not at all: 52.7, a little: 51.4, moderately: 50.5, mostly: 49.7, totally: 48.2). Moreover, compared with other types of disability (49.7 - 50.7), PWD with multiple disability (46.1) had the most negative attitude toward disability (Table 3).

## Discussion

This study was the first of its kind to use the global and cross-population ADS scale to evaluate attitudes toward disability among PWD, caregivers, and the public, as well as compare the levels of attitudes of PWD among three groups.

The longer caregivers cared for PWD, the more negative their psychological state and attitude, thus the more negative influence on the quality of caregiving [19]. Our research showed that caregivers held less favorable attitudes toward disability than the other two groups, and, furthermore, supported earlier study findings that the longer the time caregivers cared for PWD, the more negative their attitudes were. In this study, the average duration of care-taking was 17.7 years. In other words, caregivers bore the responsibility for providing personal assistance to PWD for one third of their lifetime. In China, almost all caregivers of PWD are PWD family members, and most of them are PWD’s direct relatives [33, 34]. Our study also showed that over 90 % of caregivers were family members, and more than half of them were parents/children of PWDs. In China, family caregivers provide uncompensated care to the PWD along with emotional support, and with tangible support that includes the performance of physical living tasks. Mastering these tasks and providing the timely and emotional support for PWD is a challenge for caregivers, and numbers of researchers have reported that caregivers had higher levels of depression, anxiety, and guilt [35–37]. Given the increased strains on caregivers in China, these psychological levels may particularly impact caregivers’ negative attitudes toward disability and PWD. For this situation, government support for PWD’s family is needed. However, most government financial support policy for PWD in China is based on the family unit, known as the “PWD-family” or those families having family members with disabilities. Social assistance and subsidies are usually only provided to eligible PWD-families, and not to individual persons with a disability [33]. In addition, generally the criteria for PWD-family eligibility depends upon stringent economic conditions, and financial support would be provided, for example, “only for [the] lowest level income PWD-family” [33, 38]. As a result, government provides financial support to a PWD-family only when PWDs have very limited financial support from their family. The majority of family caregivers are not eligible for government support, and therefore usually shoulder PWD’s costs of living expenses and therapy for several years, as well as devote significant amounts of time to care-taking [33]. All of these burdens may influence caregivers’ attitudes toward disability and PWD. As Chinese policy and law have bound caregivers to PWD in the unit of a PWD-family to financially and physically support PWD, this family-based care model, while obviating a financial burden for the government, results in overwhelming financial and mental pressures on caregivers [39], thus influencing the quality of PWD care [19]. Studies to evaluate the burden of PWD-family diseases due to disability among both PWD and caregivers in China should be undertaken to determine the actual extent of the burden. Moreover, with the speed of economic growth in the country, a new health-care or social insurance model is called for, one which is able to reduce catastrophic costs for PWD individuals and their families.

In contrast to our expectation, PWD, compared with the other two groups, hold the most favorable attitudes toward disabilities. The possible reason for PWD’s more positive attitudes is that most PWD in our study had been disabled for more than 10 years and perhaps had adapted to their disabilities and generally accepted them after years of living with them. Previous studies verified that positive attitudes toward disability are associated with greater acceptance of disability in oneself [22]. Therefore, as in the results, the longer the duration of disability, the more tolerant and accepting a PWD may be of his/her disability. The results also imply that PWD with shorter durations of disability hold more negative attitudes compared with longer ones, and that these individuals need mentoring or support to assist them in coping and adapting to their disability.

Regarding results for the public, this group’s *Discrimination and Gains* were the most negative domains compared with PWD and caregivers, indicating that discrimination toward disabled persons is a common phenomenon based on the public’s perception, and also that the public perceives that PWD may not be able to achieve and gain as much as those without disabilities. Public discrimination toward disability is well documented in developed countries [8]. Because “disability is viewed as a punishment for the disabled person’s sins in a past life or the sins of the person’s parents” [14, 15] in traditional Chinese culture, Chinese people may demonstrate less compassion for PWD and fail to accept or appreciate the need for integration of PWD into society. Given the prevalence of the belief, anti-discrimination campaigns for PWD will need to take this factor into consideration.

Older age was also associated with negative attitudes toward disability among PWD, caregivers, and the public. With the increase in life expectancy in most countries in the world, the challenge of ageing populations with disability has become a pressing global health issue [40]. Elders with disability suffer not only disability but also the health-related problems caused by ageing, including physical, psychological, and social function degeneration [41]. As our results indicated, elder caregivers, most of whom are PWD’s parents, must provide both physical and financial support for their disabled children, which becomes more demanding and difficult as ageing occurs. Without the capacity for independent living, PWD in China are cared for by their parents for several years and/or throughout their lifetime. The results suggest that ageing exhibits a negative impact on the attitudes toward disability and PWD. Therefore, in policy-making and in medical research, more focus should be placed on elderly PWD and caregivers, who make up the vulnerable subgroups among the disability-related population.

Finally, this study had a number of limitations. First, because it was a cross-sectional study, an observed association cannot be interpreted as causality. Second, the present study was limited to Guangzhou permanent residents and did not include other groups such as migrants from other cities. Finally, among this sample, many participants reported a long duration of years of disability; thus, their attitude toward disability may differ from that of people newly experiencing a disability. Further research needs to be conducted on larger samples of the population to include migrants and more newly-disabled persons.

## Conclusions

Acknowledging its limitations, this is nonetheless one of the first studies that has applied the cross-population ADS among both people with disability and healthy respondents. It provides valuable information about the different levels of attitudes toward disability among three groups in China. Under the PWD-families care model in China, evidence suggests that PWD’s family-member caregivers who bear major financial, emotional, and psychological burdens related to disability need more empowerment. The current model may deteriorate caregivers’ wellbeing and also undermine PWD’s independent living capacity. The development of an improved health-care model for PWD is essential. A suggested model would replace the current PWD-family unit base in which government social assistance and subsidies for disabled persons’ requirement is based on the low income level of PWD’s family. A revised model would use PWD’s individual situations as a unit base to alleviate some of the burden on family caregivers, and thus lead to positive behaviors for PWD’s caregiving and to improved quality life for PWD.

## Abbreviations

ADS: Attitude to Disability Scale
DPC: Disabled Person Card
DPF: Disabled Persons’ Federation
MDG: Millennium Development Goals
PWD: People with disability
WHO: World Health Organization
